# Changes in Microbial Foodborne Pathogen Detection in Beijing, China, Before and After the COVID-19 Pandemic: Single-Center Retrospective Study

**DOI:** 10.2196/96940

**Published:** 2026-09-21

**Authors:** Chao Liang, Luping Tan, Hongyi Zhang

**Affiliations:** 1 Department of Infectious Diseases, Peking University Third Hospital, No. 49, Huayuan North Road, Haidian District, Beijing, 100191, China, 86 15210753436

**Keywords:** descriptive analysis, etiology, epidemiology, microbial foodborne pathogens, diarrheagenic *Escherichia coli*, COVID-19 pandemic, single-center study

## Abstract

**Background:**

Foodborne diseases remain a significant global public health concern, imposing substantial health and economic burdens. The COVID-19 pandemic altered health care–seeking behaviors, infection control practices, and infectious disease epidemiology; however, its association with microbial foodborne pathogens remains incompletely characterized.

**Objective:**

This single-center retrospective study aimed to analyze changes in bacterial and viral foodborne pathogen detection using clinical and etiological data from patients at an infectious diarrhea clinic in a tertiary Beijing hospital over 6 years and to inform prevention strategies in the postpandemic era.

**Methods:**

We collected data from patients presenting to the Infectious Diarrhea Clinic of Peking University Third Hospital (a grade A tertiary care center serving the Haidian District and surrounding areas of Beijing) from January 2018 to December 2019 (prepandemic) and from January 2022 to December 2023 (postpandemic). The years 2020 and 2021 were excluded due to major disruptions in clinical services and surveillance during the peak pandemic period. Etiological results were obtained from the Beijing Haidian District Center for Disease Control and Prevention. Diagnostic methods, laboratory protocols, specimen collection procedures, reagent kits, instrumentation, and testing criteria remained consistent throughout the study period. Cases were divided into 2 groups based on time period, and *χ*2 tests were used for comparisons. The positivity rate was calculated as patients with at least 1 target pathogen detected divided by all patients who provided stool specimens.

**Results:**

In total, 1064 patients were included (561 in group A and 503 in group B). From 2018 to 2019, 155/561 specimens tested positive for foodborne pathogens (27.6%, 95% CI 23.9%‐31.3%), including 22 *Salmonella*, 24 *Vibrio parahaemolyticus*, 66 diarrheagenic *Escherichia coli* (DEC), and 43 *Norovirus* cases. From 2022 to 2023, 82/503 specimens tested positive (16.3%, 95% CI 13.1%‐19.5%), including 13 *Salmonella*, 7 *V parahaemolyticus*, 45 DEC, and 17 *Norovirus* cases. The overall positivity rate was significantly lower in the postpandemic period than in the prepandemic period (27.6% vs 16.3%, *P*<.05). Students and males aged 16‐25 years were the most represented demographic groups. DEC remained the predominant pathogen in both periods. The detection rates of *V parahaemolyticus*, from 24/561 (4.3%) to 7/503 (1.4%) (*P*=.005), and *Norovirus*, from 43/561 (7.7%) to 17/503 (3.4%) (*P*=.002), were significantly lower in the postpandemic period.

**Conclusions:**

Among patients attending a single infectious diarrhea clinic in Beijing, the detection rates of microbial foodborne pathogens were significantly lower in the postpandemic period compared with the prepandemic period. These findings may reflect changes in health care–seeking behavior, infection control practices, food consumption patterns, and/or testing practices during this period; however, this observational study cannot establish causation. Targeted surveillance and health education for students and young adults remain important. These results may not be generalizable to community-level incidence or other settings; further multicenter studies are warranted.

## Introduction

Foodborne diseases comprise a category of infectious or toxic conditions caused by various pathogenic factors that enter the human body through food intake [1]. These diseases not only cause physical harm but also affect the development of national food safety industries and impose substantial economic burdens, both of which are receiving increasing attention. Since 2010, China has established a national foodborne disease reporting system and an outbreak reporting system [2]. According to the World Health Organization, approximately one-tenth of the global population suffers from foodborne microbial infections annually, placing a heavy burden on public health [3]. Over the past decade, China has recorded over 30,000 foodborne disease outbreaks involving nearly 260,000 cases, and an estimated 200 million people are affected each year [4].

Foodborne diseases have become a key focus in China. Since 2020, the novel coronavirus has continued to spread and evolve across more than 200 countries and regions. During the COVID-19 pandemic, increases in respiratory syncytial virus infections in Canada [5], influenza infections [6], and bloodstream infections in Europe and the United States [7,8] have been reported. Studies have also described significant changes in bacterial colonization patterns during the pandemic, which may pose potential public health risks [9]. In addition, bacterial infections caused by *Streptococcus pneumoniae*, *Haemophilus influenzae*, and *Mycoplasma pneumoniae* have increased following the pandemic [10].

Assessing the association between the COVID-19 pandemic and other infectious diseases is crucial for infection prevention and control. Several studies have documented changes in gastrointestinal infections during the pandemic, with many reporting reductions in enteric pathogen detection coinciding with nonpharmaceutical interventions, such as hand hygiene, mask wearing, and social distancing. However, most existing studies have focused on Western populations, and data from hospital-based settings in China remain limited, particularly regarding the postpandemic period of 2022‐2023. This single-center retrospective study aimed to analyze changes in the detection patterns of bacterial and viral foodborne pathogens by conducting an in-depth analysis of clinical and etiological data from patients attending an infectious diarrhea clinic at a tertiary hospital in Beijing over a 6-year period and to provide evidence to inform prevention strategies in the postpandemic era.

## Methods

### Study Setting and Data Collection

This retrospective study was conducted at the Infectious Diarrhea Clinic of Peking University Third Hospital, a large grade A tertiary care hospital located in Haidian District, Beijing. The clinic serves a diverse population, including local residents, university students from surrounding academic institutions, and referred patients from the community. It functions as a primary and a secondary care access point, as well as a tertiary referral center. Data were collected from patients treated for foodborne diseases during two periods: January 2018 to December 2019 (group A, prepandemic) and January 2022 to December 2023 (group B, postpandemic).

The years 2020 and 2021 were excluded from the analysis because they represent the peak of the COVID-19 pandemic in Beijing, during which routine infectious diarrhea clinic services were substantially disrupted, surveillance protocols were altered, and patient care-seeking patterns were profoundly affected. We acknowledge that excluding this period creates a discontinuity in the temporal trends and limits our ability to capture changes during the most intense phase of the pandemic; however, data from this interval were considered unreliable for comparative analysis because of these major disruptions.

### Inclusion Criteria

Inclusion criteria were patients aged 16 years or older presenting to the Infectious Diarrhea Clinic and having illness onset associated with food intake or suspected food intake, accompanied by gastrointestinal symptoms, including nausea, vomiting, abdominal pain, and diarrhea.

Exclusion criteria were patients with gastrointestinal diseases clearly diagnosed as nonfoodborne. This study focused exclusively on bacterial and viral foodborne pathogens; nonmicrobial foodborne illnesses, such as those caused by chemical contamination, naturally occurring toxins, or food allergies, were not included in the etiological testing panel and therefore are not represented in these results.

### Laboratory Methods

For patients visiting the Infectious Diarrhea Clinic, demographic information (name, age, occupation), clinical symptoms (abdominal pain, diarrhea, fever, fatigue), suspected food exposure history, and stool specimens were collected. Specimens were sent weekly to the Haidian District Center for Disease Control and Prevention for testing, with results reviewed by the Beijing Center for Disease Control and Prevention. Importantly, the diagnostic methods, laboratory protocols, specimen collection procedures, reagent kits, instrumentation, and testing criteria remained unchanged throughout the study period (2018‐2023), with no modifications made to the pathogen testing panel or definitions of positive results.

Laboratory analyses followed the *National Foodborne Pathogen Monitoring Manual* for isolation, culture, and identification (2015‐2019). Initial screening involved streaking specimens onto agar plates with 24-hour incubation. For positive screens, 5 suspicious diarrheagenic *Escherichia coli* (DEC) colonies were selected and subjected to biochemical identification using a VITEK2 Compact30 automated system (bioMérieux Inc).

The 5 target pathogens monitored were *Salmonella*, *Shigella*, *Vibrio parahaemolyticus*, DEC, and *Norovirus*. A specimen was considered etiologically positive if at least 1 of these 5 pathogens was detected. The positivity rate was calculated as the number of positive specimens divided by the total number of patients whose stool specimens were collected and submitted for testing.

### Ethical Considerations

This study was approved by the Medical Scientific Research Ethics Committee of Peking University Third Hospital (2024 Medical Ethics Review number 203‐01). All study participants provided informed consent. The study was conducted in accordance with the Declaration of Helsinki.

### Statistical Analysis

Data were organized and summarized using Microsoft Excel. Statistical analyses were performed using SPSS version 26.0 for Mac (SPSS Inc). Cases were divided into 2 groups: group A (2018‐2019) and group B (2022‐2023). Age was compared between groups using independent-samples *t* tests. Qualitative data were expressed as numbers (percentages), and 95% CIs were calculated for key proportions. Group comparisons were made using *χ*^2^ tests, with *P*<.05 considered statistically significant.

## Results

### General Information

From January 2018 to December 2019 and from January 2022 to December 2023, a total of 1064 cases meeting the inclusion criteria were seen at the Infectious Diarrhea Clinic. Among them, 561 (52.7%) were male and 503 (47.3%) were female. The predominant age group was 26 to 45 years (n=479, 45%). The top 3 occupations were students (n=377, 35.4%), civil servants (n=284, 26.7%), and retired personnel (n=101, 9.5%). Table 1 presents a comparison of the demographic and occupational characteristics of groups A and B. Clinical symptoms among the cases are summarized in Table 2. The digestive system was the most commonly affected system in both groups (group A: 554/561, 98.8%; group B: 496/503, 98.6%). General symptoms were significantly more frequent in the prepandemic group (group A: 176/561, 31.4%; group B: 62/503, 12.3%; P<.001), while respiratory, cardiovascular/cerebrovascular, urinary, and nervous system symptoms were rare in both groups.

**Table 1.** Demographic and occupational characteristics of the study population (N=1064).

Characteristic	Overall (N=1064), n (%)	Group A (N=561), n (%)	Group B (N=503), n (%)
Gender			
Male	561 (52.7)	295 (52.6)	266 (52.9)
Female	503 (47.3)	266 (47.4)	237 (47.1)
Age group			
16‐25 years	411 (38.6)	198 (35.3)	213 (42.3)
26‐45 years	479 (45)	274 (48.8)	205 (40.8)
46‐65 years	118 (11.1)	64 (11.4)	54 (10.7)
>65 years	56 (5.3)	25 (4.5)	31 (6.2)
Occupation			
Student	377 (35.4)	180 (32.1)	197 (39.2)
Civil servant	284 (26.7)	167 (29.8)	117 (23.3)
Retired	101 (9.5)	47 (8.4)	54 (10.7)
Homemaker/unemployed	35 (3.3)	28 (5)	7 (1.4)
Worker/migrant worker	19 (1.8)	16 (2.9)	3 (0.6)
Teacher	16 (1.5)	9 (1.6)	7 (1.4)
Food service	7 (0.7)	6 (1.1)	1 (0.2)
Medical staff	11 (1)	9 (1.6)	2 (0.4)
Farmer/fisherman	3 (0.3)	2 (0.4)	1 (0.2)
Other/unknown	211 (19.8)	97 (17.3)	114 (22.7)

**Table 2.** Distribution of clinical symptoms among cases.

Symptom system	Group A (N=561), n (%)	Group B (N=503), n (%)
Digestive system	554 (98.8)	496 (98.6)
General symptoms	176 (31.4)	62 (12.3)
Respiratory system	2 (0.4)	0 (0)
Cardiovascular/cerebrovascular	2 (0.4)	0 (0)
Urinary system	1 (0.2)	0 (0)
Nervous system	5 (0.9)	3 (0.6)

### History of Suspected Food Exposure

Table 3 shows that mixed foods accounted for the highest proportion in both groups, followed by meat and meat products. *χ*^2^ tests revealed statistically significant differences between the 2 groups in the proportions of cases associated with vegetables and their products, grains and their products, and other foods (*P*<.05). The number of cases related to vegetables and their products decreased from 79 to 41 in the postpandemic period, while cases related to grains and their products increased from 24 to 47, and those related to other foods increased from 56 to 70.

**Table 3.** Suspected food exposure history among foodborne disease cases.

Food category	Overall (N=1064), n (%)	Group A (N=561), n (%)	Group B (N=503), n (%)	Chi-square (*df*)	*P* value
Meat and products	217 (20.4)	125 (22.3)	92 (18.3)	2.6（1）	.11
Vegetables and products	120 (11.3)	79 (14.1)	41 (8.2)	9.3（1）	.002
Mixed foods	378 (35.5)	196 (34.9)	182 (36.2)	0.2（1）	.67
Fruits and products	78 (7.3)	36 (6.4)	42 (8.4)	1.5（1）	.23
Grains and products	71 (6.7)	24 (4.3)	47 (9.3)	10.9（1）	<.001
Aquatic products	74 (7)	45 (8)	29 (5.8)	2.1（1）	.15
Other Foods	126 (11.8)	56 (10)	70 (13.9)	3.9（1）	.047

### Etiological Results

**Figure 1.**
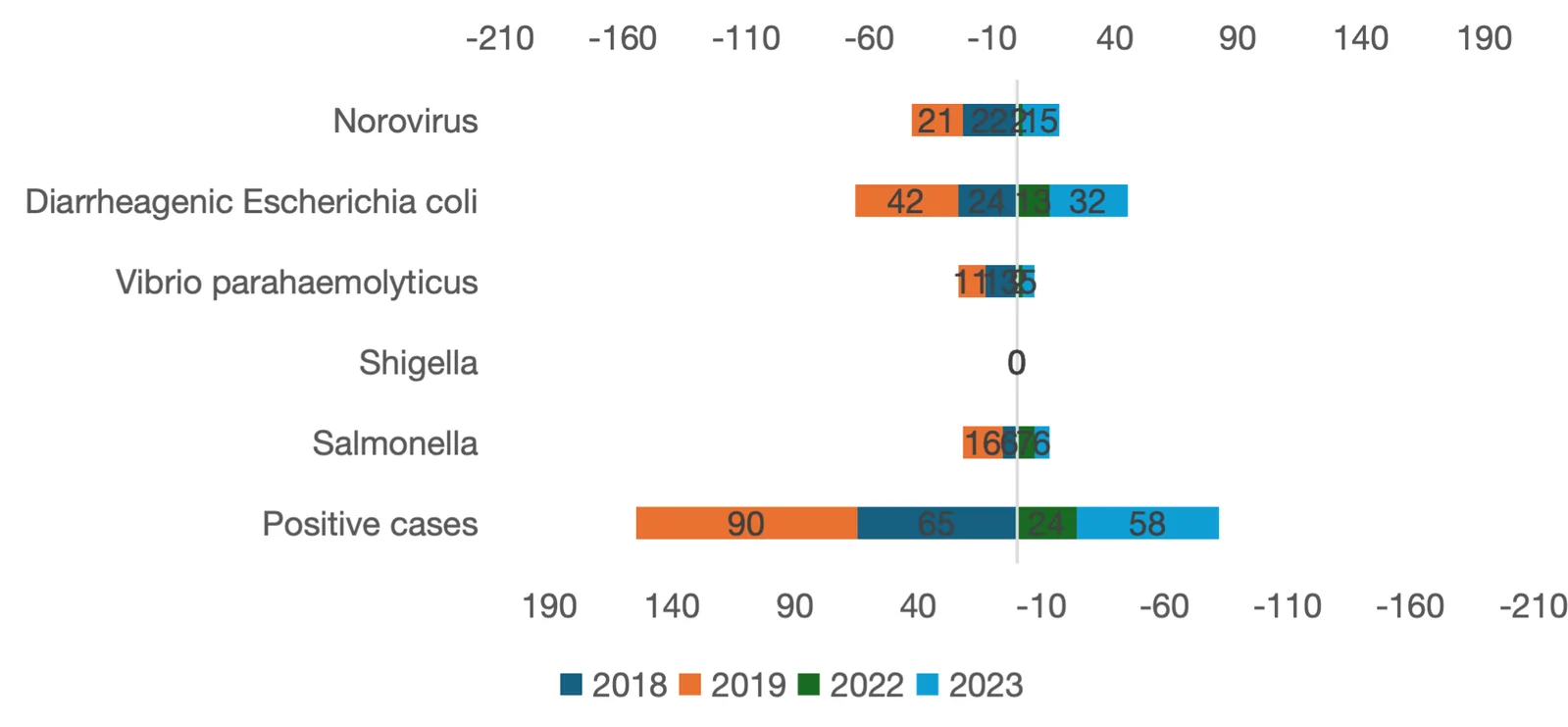
Comparison of etiological results between group A and group B of foodborne diseases.

Figure 1 compares the number of positive cases for each of the 5 monitored pathogens between the 2 periods. The overall pathogen positivity rate among all tested patients was 22.3% (237/1064). Group A had a positivity rate of 27.6% (155/561, 95% CI 23.9%‐31.3%), which was significantly higher than the 16.3% (82/503, 95% CI 13.1%‐19.5%) observed in group B (*P*<.05). Annual surveillance data showed the following results: 2018 (228 tested, 65 positive), 2019 (333 tested, 90 positive), 2022 (259 tested, 24 positive), and 2023 (244 tested, 58 positive).

Five pathogens were monitored: *Salmonella*, *Shigella*, *V parahaemolyticus*, DEC, and *Norovirus*. No *Shigella* cases were detected in either group. The detection rate of *V parahaemolyticus* among all tested patients was significantly lower in group B (7/503, 1.4%) than in group A (24/561, 4.3%) (*P*=.005). No statistically significant differences were observed for *Salmonella* (group A: 22/561, 3.9%; group B: 13/503, 2.6%) or DEC (group A: 66/561, 11.8%; group B: 45/503, 9%). The detection rate of *Norovirus* was significantly lower in group B (17/503, 3.4%) than in group A (43/561, 7.7%) (*P*=.002) (Table 4).

**Table 4.** Comparison of etiological results between group A and group B.

Item	Cases (N=1064), n	Proportion (%)	Group A (N=561), n (%)	Group B (N=503), n (%)	Chi-square (*df*)	*P* value
Positive cases^a^	237	22.3	155 (27.6)	82 (16.3)	19.7（1）	<.001
*Salmonella*	35	3.3	22 (3.9)	13 (2.6)	1.5（1）	.22
*Shigella*	0	0	0	0	0（1）	0
*Vibrio parahaemolyticus* ^a^	31	2.9	24 (4.3)	7 (1.4)	7.8（1）	.005
Diarrheagenic *Escherichia coli*	111	10.4	66 (11.8)	45 (9)	2.3（1）	.13
*Norovirus* ^a^	60	5.6	43 (7.7)	17 (3.4)	9.2（1）	.002

a Indicates statistical significance (*P*<.05).

**Figure 2.**
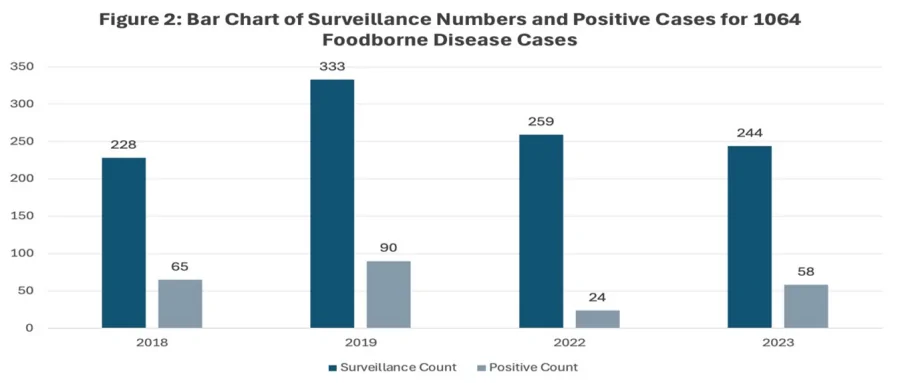
Bar chart of surveillance numbers and positive cases in 1064 foodborne disease cases.

Stacked bar charts show the number of positive cases for each of the 5 monitored pathogens in group A (2018‐2019) and group B (2022‐2023). *Shigella* had 0 positive cases in both periods. A notable decline in *V parahaemolyticus* positivity was observed in the postpandemic period. DEC remained the predominant pathogen in both periods (Figure 2).

The chart illustrates the total number of patients tested (light blue) and the corresponding number of etiologically confirmed positive cases (dark blue) for each study year. The years 2020‐2021 were excluded due to major disruptions in clinical services and surveillance during the peak COVID-19 pandemic period. This exclusion represents an important limitation, as it precludes observation of pathogen trends during the period when infection control measures were most intense.

## Discussion

### Principal Findings

This single-center retrospective study analyzed clinical and microbiological data from 1064 patients presenting to an infectious diarrhea clinic at a tertiary hospital in Beijing and compared microbial foodborne pathogen detection between the prepandemic (2018‐2019) and postpandemic (2022‐2023) periods. The principal finding was that the overall pathogen positivity rate among tested patients was significantly lower in the postpandemic period (82/503, 16.3%) compared with the prepandemic period (155/561, 27.6%), with *V parahaemolyticus* and *Norovirus* showing significant reductions. These findings are consistent with, but do not establish, a temporal association between the COVID-19 pandemic and changes in enteric pathogen detection.

### Interpretation of Findings and Comparison With Existing Literature

Among the 1064 cases, the predominant demographic comprised individuals aged 16 to 45 years (n=890, 83.6%), with students, civil servants, and retired personnel accounting for 762 (71.6%) cases. This pattern likely reflects the hospital’s location, which is surrounded by academic and research institutions. In terms of clinical characteristics, the study population was predominantly aged 16 to 25 years, with students comprising the largest occupational group. Chen et al [11] similarly reported that individuals testing positive were mainly young and middle-aged adults, highlighting the need for vigilance in monitoring students and other congregate populations.

The proportion of patients aged 26 to 45 and 46 to 65 years differed between group B and group A; however, because group B represents the postpandemic period (2022‐2023) rather than the strict lockdown period, this difference likely reflects a combination of factors, including altered health care–seeking behavior, changes in the clinic’s catchment population, differences in occupational composition, and other unmeasured variables rather than pandemic-related mobility restrictions.

In the etiological analysis, DEC remained the most frequently detected pathogen in both periods (group A: 66/561, 11.8%; group B: 45/503, 9%) [12-14], which is consistent with the findings of Liu et al in Haidian District [15], who reported an 11.8% proportion of DEC. The persistently high detection of DEC may be related to its environmental resilience, low infectious dose, and potential for foodborne transmission through contaminated produce and other food vehicles. The relative stability of *Salmonella* detection rates between periods (group A: 22/561, 3.9%; group B: 13/503, 2.6%) is consistent with its endemic circulation in animal reservoirs and continuous food supply chain contamination, which may be less affected by personal hygiene measures [16,17].

The significant reduction in *V parahaemolyticus* detection, from 24/561 (4.3%) to 7/503 (1.4%), is noteworthy [18,19]. This pathogen is classically associated with seafood consumption, particularly raw or undercooked shellfish. The observed decline may reflect changes in food consumption patterns during and after the pandemic, including reduced restaurant dining, increased home cooking, and possible disruptions in seafood supply chains. Additionally, *V parahaemolyticus* has a summer seasonal peak, and changes in health care–seeking behavior for mild diarrhea during summer months may have contributed to the observed reduction. *Norovirus* detection also declined significantly, from 43/561 (7.7%) to 17/503 (3.4%) (*P*=.002), which is biologically plausible given that *Norovirus* can be transmitted through the fecal-oral route and aerosols; thus, enhanced hand hygiene, mask wearing, and environmental disinfection measures implemented during the pandemic may have reduced its transmission [20,21].

No *Shigella* cases were identified in either group, which is consistent with the findings of Wang et al [22], who reported a gradual decline in *Shigella* among foodborne pathogens, and with Lu et al [23], who noted high *Norovirus* detection rates among viral pathogens and high DEC detection rates among bacterial pathogens. Among cases of foodborne illness, the relative proportions of *Salmonella* and DEC were higher in group B than in group A, suggesting that these pathogens may require continued surveillance.

### Alternative Explanations and Limitations

It is critical to distinguish between a reduction in detected cases and a true reduction in population-level disease incidence. This hospital-based study cannot establish population-level incidence. The observed lower positivity rate may reflect multiple factors beyond a true decline in disease occurrence, including (1) changes in health care–seeking behavior, whereby patients with milder symptoms may have been less likely to visit the hospital during and after the pandemic; (2) altered health care accessibility and clinic volume; (3) changes in food consumption patterns, including increased home food preparation and reduced dining out; (4) changes in stool sampling practices and clinician testing thresholds; and (5) nonpharmaceutical interventions (hand hygiene, masking, social distancing) that may have genuinely reduced the transmission of enteric pathogens. We are unable to disentangle the relative contributions of these factors in this observational study.

Several additional limitations should be acknowledged. First, this was a single-center study conducted at one infectious diarrhea clinic in a tertiary hospital in Beijing; therefore, the findings may not be representative of the general population or community-level epidemiology in China or in other geographic regions. This study included only symptomatic patients seeking care, which likely excluded milder cases and asymptomatic infections. Second, the exclusion of the 2020‐2021 time period represents a major gap, as this period encompassed the peak of the pandemic, when infection control measures were most intense. This created a discontinuity in temporal trends and meant that we could not characterize changes during this period. Third, we investigated only 5 bacterial and viral pathogens; nonmicrobial foodborne illnesses (chemical contamination, natural toxins, food allergies, and parasites) were not assessed, and thus our results do not represent the full spectrum of foodborne diseases. Fourth, statistical analyses were limited to univariate comparisons, and we were unable to perform multivariate adjustment for all potential confounders. Fifth, the sample size for some individual pathogens was small, limiting statistical power for subgroup comparisons. Finally, we did not have data on total clinic visit volumes to directly quantify changes in health care utilization.

### Implications

In clinical practice, early management of suspected foodborne cases, particularly those involving DEC and *Salmonella*, should include appropriate infection control measures, such as hand hygiene, use of masks and gowns, patient education on personal hygiene, environmental disinfection, vital sign monitoring, and dietary guidance. For severe cases, temporary fasting and nutritional support may be required [24,25]. Future efforts should strengthen foodborne disease surveillance through diversified health education channels and coordinated online and offline public awareness campaigns to improve food safety knowledge.

### Conclusions

Among patients presenting to a single infectious diarrhea clinic in Beijing, detection rates of bacterial and viral foodborne pathogens were significantly lower in the postpandemic period (2022‐2023) compared with the prepandemic period (2018‐2019), with *V parahaemolyticus* and *Norovirus* showing significant declines. These temporal changes may be associated with infection control measures, shifts in health care–seeking behavior, and changes in food consumption patterns; however, this observational study cannot establish causation, and the results do not represent population-level incidence or the full spectrum of foodborne diseases. Continued surveillance and further multicenter studies incorporating longer study periods and molecular subtyping are needed to better understand the long-term implications of the pandemic for foodborne disease epidemiology.

## Abbreviations

DEC: Diarrheagenic *Escherichia coli*
